# Supplementary material for: Social Isolation, Hospitalization, and Deaths from Cardiovascular Diseases during the COVID-19 Epidemic in São Paulo Metropolitan Area in 2020
Source: Int J Environ Res Public Health. 2022 Sep 2;19(17):11002. doi: 10.3390/ijerph191711002 (PMC9518093; doi:10.3390/ijerph191711002)
Supplement: Supplementary file 1 [file ijerph-19-11002-s001.zip › ijerph-1877683-supplementary.pdf]

Table S1(supplementary material): Social isolation (SI) and hospitalizations for cardiovascular disease (CVD), ischemic heart disease (IHD), and stroke from March/2020 to January/2021.

	**SI (%)**	**CVD**	**IHD**	**Stroke**
March/20	43,07	52,11	14,16	9,96
April/20	52,33	34,69	10,19	7,78
May/20	50,71	29,91	8,43	7,46
June/20	48,47	34,37	9,75	8,41
July/20	45,77	38,22	10,78	8,27
August/20	44,45	41,57	11,85	8,66
September/20	43,13	45,29	13,13	8,13
October/20	42,32	45,60	11,90	8,93
November/20	41,47	49,85	13,40	9,23
December/20	40,94	41,55	12,19	8,00
January/21	42,03	46,40	13,00	9,65
